# Factors affecting access to health services by older adults in an urban community in Thailand: a cross-sectional study

**DOI:** 10.12688/f1000research.110551.2

**Published:** 2023-02-01

**Authors:** Areeya Jirathananuwat

**Affiliations:** 1 Department of Health Technology, Faculty of Sciences and Health Technology, Navamindradhiraj University, Bangkok, 10300, Thailand

**Keywords:** Access to health service, Elderly, Urban areas

## Abstract

**Background**: The impending rapid change in Thailand’s older population has many important implications for health policy, especially older adults’ health problems, which are major cause of  them accessing health services. This study aimed to study factors affecting access to health services for older adults in urban communities in Thailand, as well as performing a situational survey of health service utilization.

**Methods:** This cross-sectional study included 886 older adults from four different neighborhoods (slum, city, suburban, and community building). Data were collected using an interview questionnaire. Information about health service variables were extracted and followed the five dimensions of accessibility by Penchansky & Thomas: availability, accessibility, accommodation, affordability and acceptability. Data were analyzed using percentage mean and standard deviation (SD). Analysis of factors affecting access to health service was performed using Logistic regression.

**Results:** The utilization of health services was high among five dimensions. There is no significant difference among other dimensions statistically from all neighborhoods. Only the acceptability dimension with notable difference (p=0.003), city neighborhood has the lowest acceptability dimension (mean 4.0, SD.=0.62), while community building has the highest one (mean 4.3, SD.=0.55). Factors affecting older adults access to health services were using health insurance rights for health care service, and concern about the necessity of health care.

**Conclusions:** Encouraging older adults to change their health insurance rights to the nearest hospital and promoting the provision of holistic health information, which will support older adults in accessing more health services to improve their health outcomes.

## Introduction

An older adult is defined by the United Nations as a person who is over 60 years of age. In 2017, the world had a population of 7.550 million people. Approximately 12.7% were older adults, meaning that our world has become an ‘aging society’. In 2017, older adults made up 17.1% of Thailand’s population, ^1^^,^^2^ and this expected to increase to 25.2% by 2030 ^3^ and 30% by 2035. ^2^ This changing population has a direct impact on public health policy and countries due older adults having different physical, mental, emotional, and social health issues to other demographics. The most common issues are related to economic, social and health issues, especially chronic health issues that require dependency from other people.

Due to chronic illnesses of older adults, health policies need to ensure that health services cover patient treatment and prevention of health issues, as well as providing a well-prepared health service system. ^4^ The World Health Organization ^5^ has suggested three concepts (factors of health, participation and security factors) to ensure that people will be covered by health services regardless of income, ethnicity gender and age. Their access to health services depends on financial factors, sufficient service and the organization of the health service. In 2002, Thailand’s government has implemented medical insurance rights ^6^ with the goal of giving people access to health care services. However, medical insurance rights do not ensure that Thai people will have access to health services completely due to the fact that their health insurance status unmatched with their service point. Even though Thailand has universal coverage policy, most individual can only access to health services at a certain medical facility where they are registered. For those who live far away from their registered medical facility have to commute to the service point, and such situation must be challenging specially for elderly. Therefore, other related factors must be considered, such as socioeconomic factors, distance from habitation to service facilities, travel expenses, and presence of reliable caretakers. ^7^

In 2013, Thailand’s urban population was 53.6% of the total population, with an increasing trend. Previous research has shown that people in the urban areas have limited access to health services. ^8^ Good health service system management is significant to older adults in allowing them to access the service thoroughly. Therefore, the Ministry of Public Health’s primary objective is to ensure that people have access to health services when they need them. Previous studies in Thailand have shown that older adults have access to health services using health insurance status through the Government (44%), the Universal Coverage Scheme (24%), or the Social Security Scheme (3%). It was also reported that use of health services was different dependent on the health insurance rights used and geographical region. ^9^ In this study, an individual’s access to and use of health services is considered by three characteristics as defined by Aday & Anderson ^10^ : a) Predisposing Factors; b) Enabling Factors; and c) Need Factors. These three factors are independent factors, affecting elderly access to health services, which comprising of five dimensions: availability, accessibility, accommodation, affordability, and acceptability. ^11^ Due to the fact that there are different types of community in Bangkok; which are slum, city, suburban, housing community, community building, and housing estate, this study only focused on slum, city, suburban, and community building. While in demographic perspectives, population in Bangkok consists largely of migrant workers and latent population as well as those who were born and rise in the capital, and these different backgrounds may affect access to health services. This study aimed to study the factors affecting access to health services for older adults in urban communities, as well as to perform a situation survey of health service utilization.

## Methods

### Study design

This cross-sectional study was conducted among older adults from four different neighborhoods (slum, city, suburban, and community building). A purposive sampling method was used to select communities. A stratified random sampling technique was used to ensure proportional numbers of sample from slum, city, suburban, and community building urban types. Given a type I error of 0.05 and 80% power, the sample size was estimated to be 1,086.

### Participants and setting

Participants in this study were older adults who responded to the survey from September to December 2018. The inclusion criteria were a) aged >60 years; b) agree to participate in research. Those unwilling to participate or did not provide consent were excluded. Convenience sampling was used to select the participants. The setting was in Bangkok, Thailand. The numbers of participants from neighborhoods of slum, city, suburban, and community building who lived in neighborhoods were 430, 432, 108, and 116 respectively. In order to contact the participants, local community leaders and community health volunteers publicly announced the project and recruited participants in the community. Data collection took place in the community’s common space.

### Questionnaire

Data were collected using an interview questionnaire – questions were read to the participants by the researcher, and the participants responded verbally. The questionnaire was based on the five dimension of accessibility developed by Penchansky & Thomas ^11^ (availability; accessibility; accommodation; affordability; and acceptability) and the three factors affecting access to health services, defined by Aday & Anderson ^10^; Predisposing Factors, Enabling Factors, and Need Factors. Predisposing Factors mean the socio-cultural characteristics of individuals that exist prior to their illness, such as age, gender, religion, education. Enabling Factors mean the logistical aspects of obtaining care, such as monthly income, health insurance status, transport to hospital, distance and time to hospital. Need Factors mean the most immediate cause of health service use, from functional and health problems that generate the need for health care services, such as self-perceived health.

The questionnaire was composed of three sections. Section 1 surveys general demographic information of the participants. Section 2 ‘Access to health service’ consisted of 11 items including items relating to Enabling Factors and Needs Factors. Section 3 ‘Opinion on healthcare service utilization’ contained 16 items: availability (4 items), accessibility (2 items), accommodation (4 items), affordability (3 items), and acceptability (3 items). A five-point Likert scale (1=strongly disagree, 2=disagree, 3=moderately, 4=agree, and 5= strongly agree) assess the 16 items. Interpretation of opinion on healthcare service utilization score in each item was as follows: 1.00 – 2.33 indicating low level access to healthcare service, 2.34 – 3.67 indicating moderate level access to healthcare service, and 3.68 – 5.00 indicating high level access to healthcare service. With minimal amount of both low and moderate levels, this study combines the two levels together as a group in order to analyze overall data via Logistic regression.

A pilot test was performed with 30 older adults who had the same characteristics as the participants, which were aged>60 years and willing to answer. The reliability of the questionnaire was tested using Cronbach’s alpha, with results of 0.9. As a result of the pilot study, the questionnaires had no change in the wording.

A copy of the questionnaire both in Thai and English can be found in the *Extended data.*

### Data analysis

Data were analyzed by using SPSS Statistics for Windows, Version 28.0 (IBM SPSS Statistics for Windows, Version 28.0. Armonk, NY: IBM Corp). Descriptive data were using percentage mean and standard deviation (SD). Analysis of factors affecting access to health service was performed using Logistic regression.

This study used the STROBE cross-sectional checklist when writing the report. ^12^

### Ethical considerations

This study was one of 12 projects in a set of ageing research projects approved by the Institutional Review Board of the Faculty of Medicine Vajira Hospital, Navamindradhiraj University (IRB No.067/60). Information about the study was explained to the participants (objectives, data collection steps, timing, and benefits of the study). After explanation, written informed consent was obtained from all participants. Upon completing the interview, each participant received 300-baht modest amount of financial compensation for their participation.

## Results

A total of 886 older adults were included in this study. Of these 70.8% were women, the average age was between 60 and 69.9 years, and most (42.3%) participants were married and lived together. In total, 58% of participants had finished primary education and most (63.8%) were currently unemployed. Almost half of participants (45.5%) had an income less than 5,000 baht.; 58% of participants stated that their income was sufficient to cover monthly bills. Most participants came from the urban neighborhoods.

In addition, 56.5% of participants reported accessing health services in the past year for an illness or health problems that required medical treatment and most of them reported that their condition was minor. More than half of respondents reported accessing health services was necessary to them with awareness that their health conditions were minimal. Most (>60%) of participants went to health facilities by themselves, and 83.6% used health services with direct health insurance. Public health facilities were the preferred health services visited.

In terms of expenses, participants reported that transportation fares were mostly below 100 baht, while 79.5% had no fees relating to medical expenses and miscellaneous fees. Most of the transport services to public health facilities were taxis, motorcycle-taxis, tricycles and vans. A total of 45% of participants visited hospitals two or three times per month. In terms of distance, most participants reported less than 5 kilometers from home to public health services, with a travel time of less than 30 minutes most reported. Most of the medical insurances were Universal Coverage Scheme. Participant demographics are shown in Table 1.

**Table 1.** Characteristics of study subjects (n=886).

	n (%)
**Gender**	
Male	259 (29.2)
Female	627 (70.8)
**Age (years)**	
60 – 69.9	473 (53.4)
70 – 79.9	291 (32.9)
≥ 80	122 (13.7)
**Status**	
Single	185 (20.9)
Married/living together	375 (42.3)
Married/living apart	81 (9.1)
Widowed	245 (27.7)
**Education**	
No educational attainment	116 (13.0)
Primary level	514 (58.0)
Secondary level	206 (23.4)
Bachelor’s degree to post graduate studies	50 (5.6)
**Employment**	
Employed	321 (36.2)
Unemployed	565 (63.8)
**Monthly income (baht.)**	
No income	181 (20.4)
<5,000	403 (45.5)
5,000 – 10,000	204 (23.0)
>10,000	98 (11.1)
**Sufficiency of income**	
Insufficient	298 (33.6)
Adequate	514 (58.0)
Assets and savings	74 (8.4)
**Debts**	
No debts	637 (71.9)
Debts	249 (28.1)
**Type of urban communities**	
Slum	327 (36.9)
City	328 (37.0)
Suburban	115 (13.0)
Community building	116 (13.1)
**Acquisition of illness that require health service visitation in last year**	
No	385 (43.5)
Yes	501 (56.5)
**Level of health problems**	
Common	537 (60.6)
Mild	142 (16.0)
Moderate	175 (19.8)
Severe	32 (3.6)
**Necessity of health care**	
Unnecessary	249 (28.1)
Necessary	637 (71.9)
**Perceived of health conditions**	
Poor	49 (5.5)
Moderate	462 (52.1)
Healthy	319 (36.0)
Perfectly healthy	56 (6.3)
**Heading to hospital accompanied by**	
Himself/herself	556 (62.8)
Spouse	64 (7.2)
Children/Lineage	219 (24.7)
Others	47 (5.3)
**Health service utilization at public health services**	
Using health insurance rights	741 (83.6)
Using others health insurance rights	145 (16.4)
**Hospital utilization (allow more than one answer)**	
Public hospital only	565 (63.7)
Private hospital only	86 (9.7)
Health center/clinic only	73 (8.2)
Pharmacy only	12 (1.3)
Public and Private hospital	23 (2.5)
Public hospital and Health center/clinic	85 (9.5)
Public hospital and Pharmacy	24 (2.7)
Unused service	16 (1.8)
**Estimated payments in access to health facilities Transportation fare (baht.)**	
No travel expenses	235 (26.5)
<100	475 (53.6)
100–500	171 (19.3)
>500	5 (0.6)
Median = 50.0; Min-Max = 0-1,000	
**Medical expense (baht.)**	
No medical expenses	704 (79.5)
<1,000	152 (17.2)
1,000–3,000	20 (2.3)
>3,000	10 (1.1)
Median=0; Min-Max=0-6,000	
**Number of hospital visitation**	
Once or twice a month	155 (17.5)
3 to 5 times a month	66 (7.4)
More than 5 times a month	1 (0.1)
Once in 2 to 3 months	400 (45.1)
Once in 4 to 5 months	10 (1.1)
Once or twice a year	51 (5.7)
Due to sickness	107 (12.1)
Never/seldom	56 (6.3)
Unknown	40 (4.5)
**Transport to the hospital (allow more than one answer)**	
Walk only	76 (8.5)
Private car only	142 (16.0)
Bus only	141 (15.9)
Taxi/Motorcycle-taxi/tricycle/van only	349 (39.3)
Any of the 2 transport vehicles as walk or private car	122 (13.7)
**Distance from home to public health services or hospital approximately (km.)**	
<5	535 (60.4)
5–10	250 (28.2)
>10	101 (11.4)
**Travel time from home to public health services or hospital approximately (min.)**	
<30	675 (76.2)
30–59	180 (20.3)
60–120	31 (3.5)
**Health insurance status (Allow more than one answer)**	
Universal coverage scheme only	546 (61.6)
Government/State enterprise only	110 (12.4)
Social security scheme only	62 (6.9)
Self-payment	25 (2.8)
Others	85 (9.5)
Using two medical rights	27 (2.9)
Unknown rights	21 (2.3)

### Access to health services

Participants from all neighborhoods reported that access to health services was high among all five dimensions. Apart from the acceptability dimension with notable difference (p=0.003), there is no significant difference among other dimensions, statistically. City neighborhood has the lowest acceptability dimension (mean 4.0, SD.=0.62), while community building has the highest one (mean 4.3, SD.=0.55) as the findings are shown in Table 2.

**Table 2.** Access to health services of elderly from 4 types of neighborhood.

Access to health services	Slum	City	Suburban	Community building	p-value
Availability dimension	4.2 (0.60)	4.2 (0.55)	4.2 (0.63)	4.1 (0.65)	0.444
Accessibility dimension	4.1 (0.69)	4.1 (0.67)	4.1 (0.70)	4.1 (0.71)	0.607
Accommodation dimension	4.0 (0.70)	3.9 (0.64)	4.0 (0.74)	4.0 (0.56)	0.187
Affordability dimension	4.3 (0.64)	4.2 (0.58)	4.2 (0.71)	4.3 (0.58)	0.203
Acceptability dimension	4.2 (0.62)	4.0 (0.62)	4.1 (0.68)	4.3 (0.55)	0.003
**Overall**	4.1 (0.54)	4.1 (0.48)	4.1 (0.57)	4.2 (0.49)	0.286

### Factor affecting access to health service

In analyzing data on various factors, it was found that there is only data for Enabling Factors and Needs Factors that affect access to health services ( Table 3). In terms of Enabling Factors, using health insurance rights for health care service has a great impact on access to health services; those who use the rights directly at health facilities have more than 1.9 times accessibility compared with those that use rights indirectly (p-value=0.006, 95%CI=1.214–3.094). For the Needs Factors, people who are concerned and aware about their health condition tend to access health services more than those who are not (about 1.5 times odds ratio) (p-value=0.043, 95%CI=1.013–2.340).

**Table 3.** Factors affecting access to health services.

Variables	β	S.E.	Wald	p-value	Odd Ratio	95%CI
**Use of medical rights with health facilities** Directly (Using health insurance rights for health care service)	.662	.239	7.697	.006	1.938	1.214–3.094
**Necessity of health care** Necessary (Concern about the necessity of health care)	.423	.214	4.081	.043	1.540	1.013–2.340

## Discussion

This study is a part of the Health Promotion Project under the Principle for the Elderly in the Urban Area of Bangkok Metropolitan, which is influenced by a well-known national practice guideline, the Philosophy of Sufficiency Economy, initiated by King Rama IX-Bhumibol Adulyadej. Previous studies have shown that the utilization of health services by older adults in urban areas does adhere to the five dimensions as described by Penchansky & Thomas. ^11^ Firstly, based on previous data there is a high level of access to health service units at all levels from public to private sector, which are located across the country, and especially in the Bangkok Metropolis. In the Thailand’s capital city, there are a total of 4,687 health service units, and only 141 units are able to admit patients in the facility overnight. Approximately 56% of the units are in the public sector and 44% are in the private sector. ^13^ This result is consistent with previous studies ^14^^,^^15^ which explored the factors that affect access to health services of older adults in Bangkok. However, an earlier study revealed that availability of health service at national level is relatively insufficient. ^16^ Secondly, for the accessibility dimension, high level access has been shown by a previous study, ^14^ but this result is inconsistent with some studies. ^15^^,^^16^ Obstacles in accessing health services have been reported as difficulties to travel to health facilities. ^17^ However, in our study, most participants (60.4%) stated that the distance from home to health facilities was less than 5 kilometers, and it takes 76.2% of participants less than 30 minutes to travel to health facilities. This short distance enables participants to access health services easily, which are mostly public hospitals. Therefore, the participants are able to access and utilize the service conveniently.

Thirdly, for the accommodation dimension, data from our study indicates a high level of service access. A previous study ^17^ revealed that the main problem in this dimension is the long duration between making an appointment and seeing general doctors or specialists. A study by Rahman *et al*. ^18^ found that the long waiting time for doctors affected the satisfaction of patients. In addition, it was found that personal factors of patients, such as experience of previous medical care or expectation, affected satisfaction as well. ^19^ Participants in our study had high satisfaction with appointment of doctors, as they easily made appointments. Therefore, the level of accommodation for our study is at a high level access. Fourthly, for the affordability dimension, our study revealed that there is a high level of access to services. The ability to pay a medical expense is one problem in accessing health services. ^20^ The constitution of the Kingdom of Thailand B.E. 2550 ^21^ states that “Everyone has equal rights in utilization of the public health service without medical expenses and able to received public health services thoroughly and efficiently”. Accordingly, the established health insurance rights for Thai people gives everyone the opportunity to access health services equally as prices are standardized by the state. ^22^ Our study indicated that participants were satisfied with the rights and expenses at a high level because most of them used Universal Coverage Scheme rights, which has no payment. Therefore, the ability to access the service in the affordability dimension is at a high level. Fifthly, for the acceptability dimension, our study showed a high level service. A previous study ^23^ found that for this dimension service characteristics, such as attitude and expectations of service recipients, can hinder the acceptance of service quality. The concept of satisfaction of service quality is significant as patients as customers should be satisfied by the service and facility, particularly when they frequently use the health services.

In comparison, the study showed that these four different types of neighborhoods individually have mean score of access to health services slightly above four, which make no significant difference on accessing to health services. Only acceptability dimension was found different because of service mind of service providers and satisfaction of patients. Elderly from city neighborhood generally have the least acceptable services due to having no option, except public hospitals. While elderly from community building neighborhood have the most acceptability rate because they are able to access health services other than public hospitals such as private hospitals. With limited number of medical staffs in public hospitals compared to number of patients, resulting in hospital administration and human resource management, as well as satisfaction of service receivers.

This study found two factors affecting health service accessibility: a) visiting health service unit they registered at, and b) the need for the service. The former factor is aligned with Enabling Factor, the latter is aligned with Need Factor. For health insurance right factor, this study statistically found that participants that visited a health service unit that they were registered at were 1.9 times more likely to access to health service, compared to those who are not registered at the unit(95%Cl=1.214–3.094). In total 83.7% of public hospital patients visited hospitals they were registered at, which is four-times more than those who visited health service units they are not registered. This was consistent with a previous study ^15^ that found that the relationship between accessibility to health services and utilization of health services was statistically significant (p<0.05). For this reason, if patients use their rights directly the health care service is free of charge for them, or only causes minimal payment. Besides, one of factors why people access public hospitals is no medical expenses. ^24^ It’s been shown that 61.6% of older adults used Universal Coverage Scheme rights, which effects accessibility to health services. ^25^ In addition, use of treatment services increase with the use of health insurance rights, especially in outpatients. ^26^^,^^27^ The lack of ability to pay for medical expenses has been shown to be an issue with utilization of health services of older adults. ^20^

For the second factor, necessity of health care, our study revealed that participants who perceived that they needed to be treated in a hospital increases access to health facilities by 1.5 times compared to those that consider it unnecessary to be treated in a hospital (95%Cl=1.013–2.340). The necessity was making people be aware of the importance in medical treatment. However, it has been observed that using health insurance rights with no payment leads to a more accessible health services. These health services are mostly free of charge, especially for those who registered their health insurance rights at the hospital. Even though patients do not have to pay for the health services, free services are not what they actually want; usually they visit health service units due to their illness and awareness on health conditions and not just because these are free. This finding is supported by previous research ^24^ that studied the needs for access to health services from the perspective of patients and doctors. It indicated that most patients came to access services at the outpatient department; 71.8% of them thought that it was necessary to access to health services and only 0.6% thought it was unnecessary. Nonetheless the viewpoint on treatment between patients and doctors could be different; perspective is important because different perspectives lead to different decisions about necessity in treatment. A previous study ^28^ revealed the patients assumed that they knew about their health and were able to decide whether they needed treatment. Lack of knowledge of health may lead them to not taking care of themself or lead them going to hospital. While doctors are likely to suggest them to initially get diagnosed by general practitioners at health service units.

Findings from our study suggested that the government should give the opportunity to get health insurance rights, so individuals can access health facilities nearest their habitation. Similarly the government should facilitate the ability to move health insurance rights to the health facility nearest their habitation. Due to the fact that receiving health services from health service units where patients do not have their health insurance causes extra payment for them. Instead, rights could be based on the use of identification card, as there are some older adults who do not have real address in urban communities as they may be renting a house etc. Medical facilities near the habitation will make it easier way to visit, with no payment, meaning that older adults could have access to more services. Both the public and private sectors should help raise awareness of basic health care, including health literacy to increase awareness of necessity for treatment. Indeed, a previous study indicated there is a correlation between health literacy and the quality of healthcare interactions. ^29^ Enhancing an understanding of the concept of health literacy will eventually increase patient participation in health care standard and quality in the country holistically. ^30^

There are a few limitations in our study, primarily the number of participants did not reach the set target due to accessibility to participants in urban communities. Also, participants were able to be contacted only via community leaders or public health volunteers for data collecting. A target number of 1,086 participants was set initially. However, this study successfully reached about 82% of the set target, 886 participants responded in the study. This study conducted on a convenience sample will have limited generalizability.

## Conclusion

Among older adults in urban Bangkok, factors affecting access to health services were using health insurance rights for health care service, and concern about the necessity of health care. Encouraging older adults to change their health insurance rights to their nearest hospital and promoting the provision of public health information, will support older adults in accessing more health services to improve their health outcomes.

## Data availability

### Underlying data

Underlying data cannot be shared as the ethical committee that approved the study stated that only aggregated data could be shared openly. In addition, this study is part of a set of research projects about ageing, which all use the same underlying dataset; sharing the dataset must be permitted by all researchers of the ageing projects.

Researchers interested in accessing the data will need to submit an official letter of request for the data to Navamindradhiraj University and will be asked to confirm that they will not violate the ethical standards of the ethical committee and protect the anonymity of the participants. Researchers can contact the corresponding author, who can facilitate this process.

### Extended data

Open Science Framework: Factors affecting access to health services by older adults in an urban community in Thailand: a cross-sectional study, https://doi.org/10.17605/OSF.IO/MTNPC. ^31^

This project contains the following extended data:

- Copy of the questionnaire in Thai with an English translation

Data are available under the terms of the Creative Commons Zero “No rights reserved” data waiver (CC0 1.0 Public domain dedication).
