# Supplementary material for: Factors affecting access to health services by older adults in an urban community in Thailand: a cross-sectional study
Source: F1000Res. 2023 Feb 1;11:467. Originally published 2022 Apr 27. [Version 2] doi: 10.12688/f1000research.110551.2 (PMC10163336; doi:10.12688/f1000research.110551.2)
Supplement: Peer review history

# Peer review: Reviewer response for version 2

**DOI:** 10.5256/f1000research.142983.r162080

**Published:** 2023-05-05

**Authors:** Chaisiri Angkurawaranon

**Affiliations:** 1 Department of Family Medicine, Faculty of Medicine, Chiang Mai University, Chiang Mai, Thailand

I am happy with the revisions made.

Is the work clearly and accurately presented and does it cite the current literature?

Partly

If applicable, is the statistical analysis and its interpretation appropriate?

Partly

Are all the source data underlying the results available to ensure full reproducibility?

Yes

Is the study design appropriate and is the work technically sound?

Yes

Are the conclusions drawn adequately supported by the results?

Partly

Are sufficient details of methods and analysis provided to allow replication by others?

No

Reviewer Expertise:

Primary care and epidmiology

I confirm that I have read this submission and believe that I have an appropriate level of expertise to confirm that it is of an acceptable scientific standard.

# Peer review: Reviewer response for version 2

**DOI:** 10.5256/f1000research.142983.r162081

**Published:** 2023-02-21

**Authors:** Krit Pongpirul

**Affiliations:** 1 Johns Hopkins Bloomberg School of Public Health, Baltimore, MD, USA; 2 Faculty of Medicine, Chulalongkorn University, Bangkok, Thailand; 3 Bumrungrad International Hospital, Bangkok, Thailand

The author satisfactorily addressed my comments and suggestions.

Is the work clearly and accurately presented and does it cite the current literature?

Partly

If applicable, is the statistical analysis and its interpretation appropriate?

Partly

Are all the source data underlying the results available to ensure full reproducibility?

Partly

Is the study design appropriate and is the work technically sound?

Yes

Are the conclusions drawn adequately supported by the results?

Partly

Are sufficient details of methods and analysis provided to allow replication by others?

Yes

Reviewer Expertise:

Health Services, Health Systems

I confirm that I have read this submission and believe that I have an appropriate level of expertise to confirm that it is of an acceptable scientific standard.

# Peer review: Reviewer response for version 1

**DOI:** 10.5256/f1000research.122173.r155676

**Published:** 2022-12-01

**Authors:** Chaisiri Angkurawaranon

**Affiliations:** 1 Department of Family Medicine, Faculty of Medicine, Chiang Mai University, Chiang Mai, Thailand

Thank you for the opportunity to review this paper. There are some comments and suggestions.

1. In the Introduction, I am a bit surprised at the low access to health services given that Thailand has universal coverage - can the authors expand just a bit more as to why it is such a low number?
2. In the Introduction, the authors mention the five dimensions of access to health services, then three individual characteristics (by Aday & Anderson). So I'm not really sure which framework the authors will use or if they are using both, how they can connect and intersect.
3. I think the authors should also clearly state the gap in the literature that they are trying to address. Are the authors trying to compare different communities?
4. As the main objective (gap) is not clearly stated, the data analysis is also a bit underdeveloped. As the authors are presenting their access to care score as categorical data (low, moderate or high), it may not be appropriate to use only mean and sd. What is the binary outcome for the logistic regression? What were the potential confounders?  
5. Would Table 1 and Table 2 be more informative if the authors could also add columns detailing each type of community?
6. For Table 3, the outcome in the logistic model has not been clearly defined. What did the models adjust for?
7. Table 4, I'm not really sure what 'use health right non-directly' means.
8. The section of the Discussion should be a summary of your main finding to answer the question. As the question was not clearly defined in the Introduction (please see comment 3), this section may need to be revised accordingly to discuss the key findings (based on the objectives identified) within the context of the literature, including a more in-depth discussion about access to health services within Thailand (for example, why the estimate differed from previous studies mentioned in the Introduction). A discussion or limitation about the generalisability of the results may also be required.
9. From reading the Discussion, it seems like the authors want to estimate access to care for Bangkok but the authors may want to consider the variation in access to care based on the types of community. 
10. In the Conclusion, the authors focus their conclusion on changing the patient's insurance rights to their nearest hospital but from my reading (based on the results and tables), most of the patients are already using their insurance right at a nearby facility so I'm not sure about this conclusion.

Is the work clearly and accurately presented and does it cite the current literature?

Partly

If applicable, is the statistical analysis and its interpretation appropriate?

Partly

Are all the source data underlying the results available to ensure full reproducibility?

Yes

Is the study design appropriate and is the work technically sound?

Yes

Are the conclusions drawn adequately supported by the results?

Partly

Are sufficient details of methods and analysis provided to allow replication by others?

No

Reviewer Expertise:

Primary care and epidmiology

I confirm that I have read this submission and believe that I have an appropriate level of expertise to confirm that it is of an acceptable scientific standard, however I have significant reservations, as outlined above.

# Response

**Published:** 2023-01-29

**Authors:** Areeya Jirathananuwat

**Affiliations:** Navamindradhiraj University, Thailand

Dear Prof Chaisiri Angkurawaranon,

 First of all, thank you for your comments and suggestions that allowed me to greatly improve the quality of the manuscript. I agree with all your comments, and I corrected it in the new version. Additionally from my analysis of the data again, I did not find the association of types of community and access to health service. In the conclusion, the results also showed as version 1.

 Thank you once again for your valuable time

 Best regards,

 Areeya

# Peer review: Reviewer response for version 1

**DOI:** 10.5256/f1000research.122173.r136381

**Published:** 2022-11-29

**Authors:** Krit Pongpirul

**Affiliations:** 1 Johns Hopkins Bloomberg School of Public Health, Baltimore, MD, USA; 2 Faculty of Medicine, Chulalongkorn University, Bangkok, Thailand; 3 Bumrungrad International Hospital, Bangkok, Thailand

Thank you very much for the opportunity to review this work. This cross-sectional survey was conducted in one purposively selected urban community in Thailand. Although the survey result was relatively old (Sep-Dec 2018), the findings could still be useful. I have several comments and suggestions for improving this work for your consideration.

 Please specify which community was purposively selected, along with its demographic and other characteristics, along with that of other general/urban/rural communities in Thailand. This would enhance the external generalizability of this study.

 The author attempted to apply a stratified random sampling approach to ensure the representativeness of 4 'groups' of older adults in one urban community. To minimize confusing word choices, consider using 'neighborhoods' to represent the subgroups and use the term 'community' only if necessary. That is, the chosen urban community in this study comprises four different neighborhoods (slum, city, suburban, and condominium). Following this advice, the sampling technique should be changed from stratified to clustered.

 Ethical considerations: I suggest changing the phrase " *cash incentive*" to 'modest amount of financial compensation'.

 Results: 886 (instead of the calculated 1,086) were included in this study. Please calculate the percentage and provide an appropriate term. I'm not sure if this should be a 'response rate' or a 'successfully reached rate' or other terms.

 Table 1 - " *Sufficiency of income*": Provide rationale and/or reference for this measure.

 Access to health services should be comparatively presented across the five domains (Table 2). For example, why was the Accommodation relatively lower than the other dimensions?

 Also, it would be more interesting to compare the findings across the four neighborhoods.

 The terms " *medical rights*" or " *health insurance rights*" might be confusing; consider replacing them with 'health insurance status' or more relevant terms.

Is the work clearly and accurately presented and does it cite the current literature?

Partly

If applicable, is the statistical analysis and its interpretation appropriate?

Partly

Are all the source data underlying the results available to ensure full reproducibility?

Partly

Is the study design appropriate and is the work technically sound?

Yes

Are the conclusions drawn adequately supported by the results?

Partly

Are sufficient details of methods and analysis provided to allow replication by others?

Yes

Reviewer Expertise:

Health Services, Health Systems

I confirm that I have read this submission and believe that I have an appropriate level of expertise to confirm that it is of an acceptable scientific standard, however I have significant reservations, as outlined above.

# Response

**Published:** 2023-01-29

**Authors:** Areeya Jirathananuwat

**Affiliations:** Navamindradhiraj University, Thailand

Dear Prof Krit Pongpirul,

 Thank you very much for reviewing my paper. Your guidance is very helpful in my study. I have revised the paper based on your suggestions.

 Best Regards,

 Areeya
